# Supplementary material for: Specific hippocampal representations are linked to generalized cortical representations in memory
Source: Nat Commun. 2018 Jun 7;9:2209. doi: 10.1038/s41467-018-04498-w (PMC5992161; doi:10.1038/s41467-018-04498-w)
Supplement: Supplementary file 1 — Supplementary Information [file 41467_2018_4498_MOESM1_ESM.docx]

**Supplementary Figures**

**Specific hippocampal representations are linked to generalized cortical representations in memory.**

**Yu et al.**

**
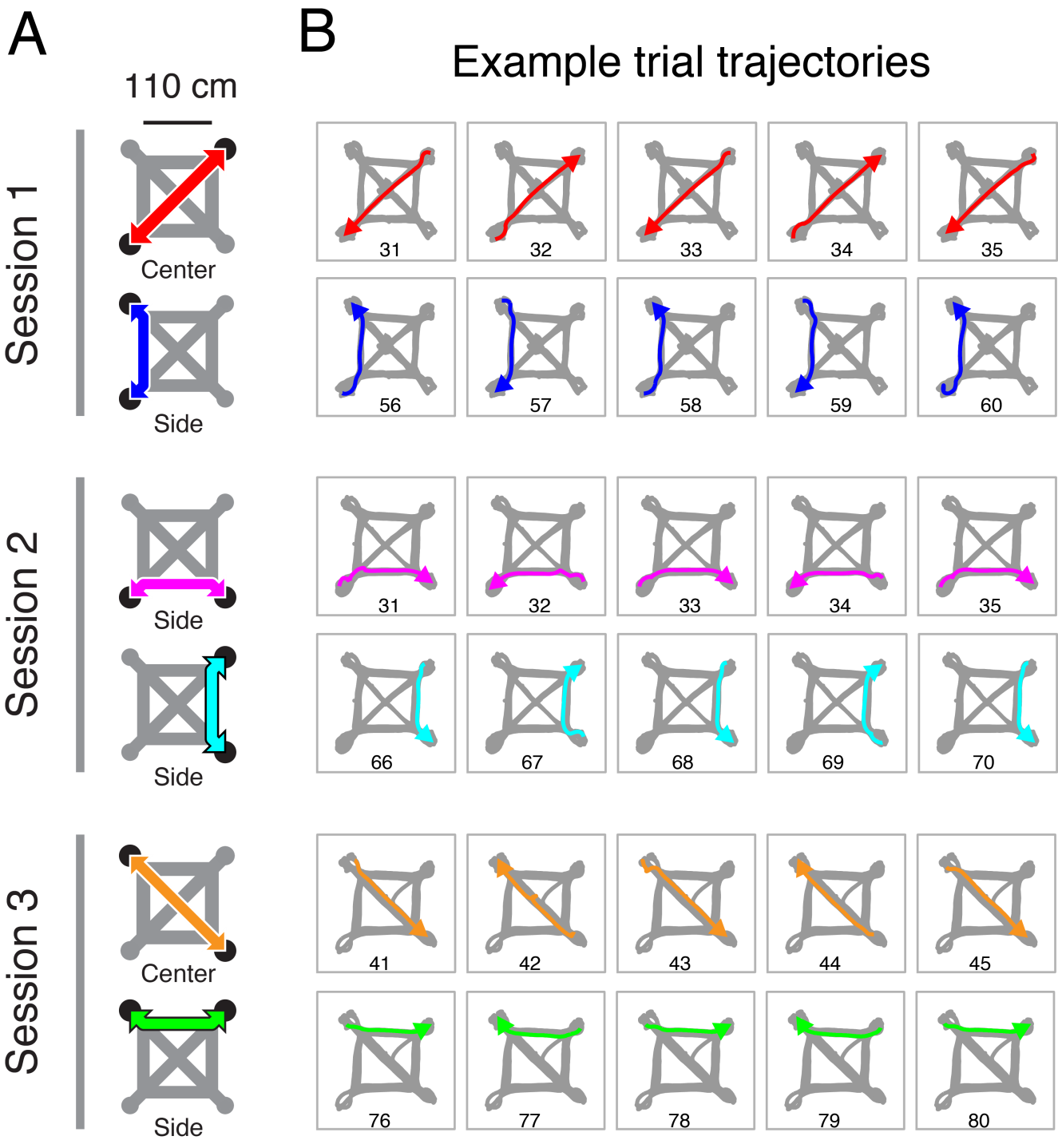
**

**Supplementary Figure 1. Task structure and example trajectories.**

**A.** Arrangement of rewarded well locations (black circles) and the most direct interconnecting path (colored arrows) in the maze (gray). The type of path is indicated below each schematic. “Center” refers to paths that cross the center of the maze. “Side” refers to paths on the sides of the maze. The reward well locations changed within and between sessions.

**B.** The rat’s trajectory on 5 consecutive trials for each reward well location contingency. The destination well is indicated by an arrow. The trial number relative to the start of each session is indicated.

**
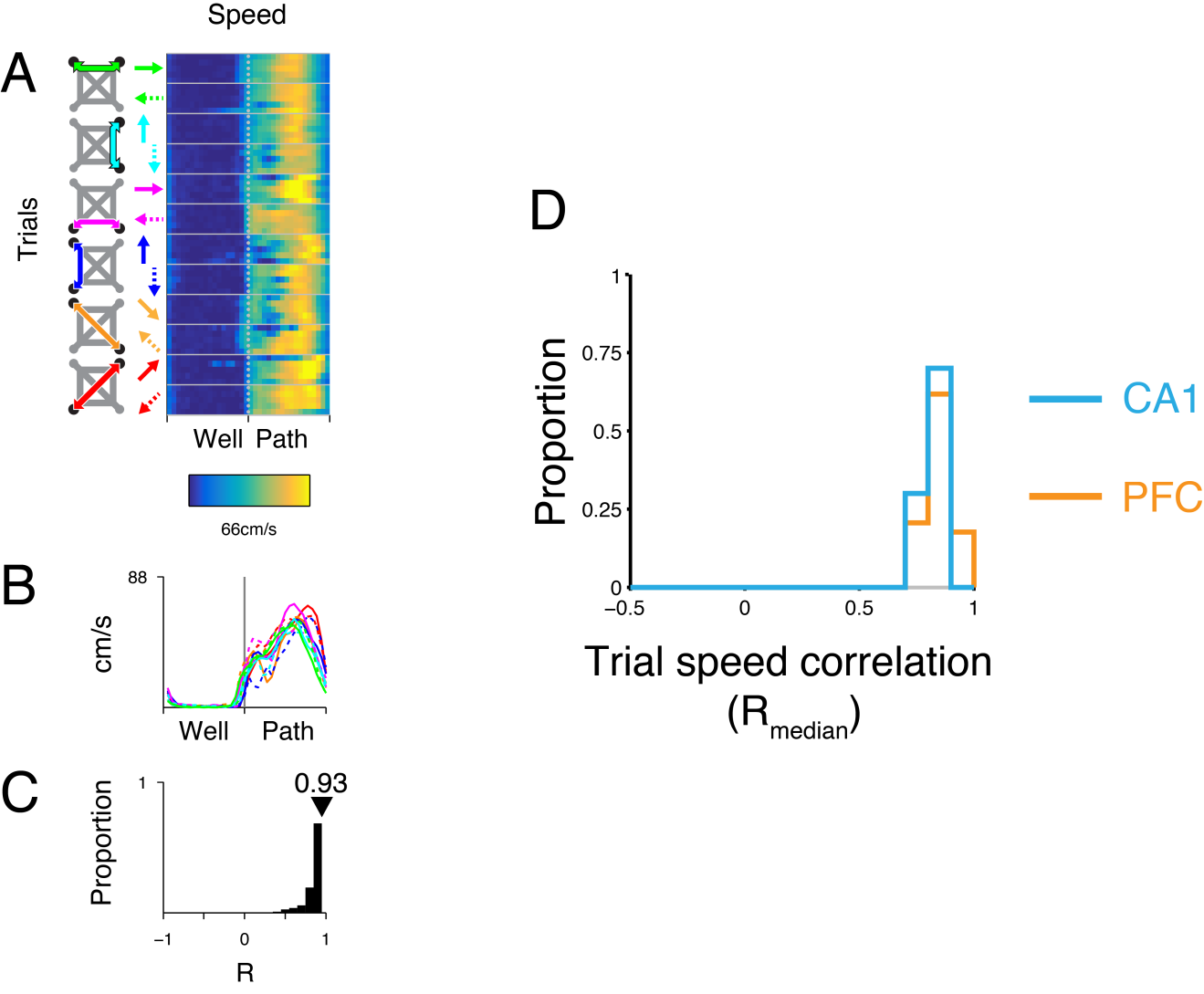
**

**Supplementary Figure 2. Speed profile similarity.**

**A.** Trial speed profile for trials shown in Fig. 1. The trajectory of each trial is indicated by the schematic on left. Solid and dotted arrows indicate opposing directions of travel on a path.

**B.** Median speed for each trajectory. Line color key correspond to arrow color scheme in A.

**C.** Distribution of Pearson’s R for pairwise trial speed profile correlations. The median of the distribution (R_median_) is indicated in red.

**D.** Trial speed profile R_median_ for trials on days used for calculating CA1 (cyan, n=10) and PFC (orange, n=35) firing similarity. Kolmogorov-Smirnov test: not significant.

**
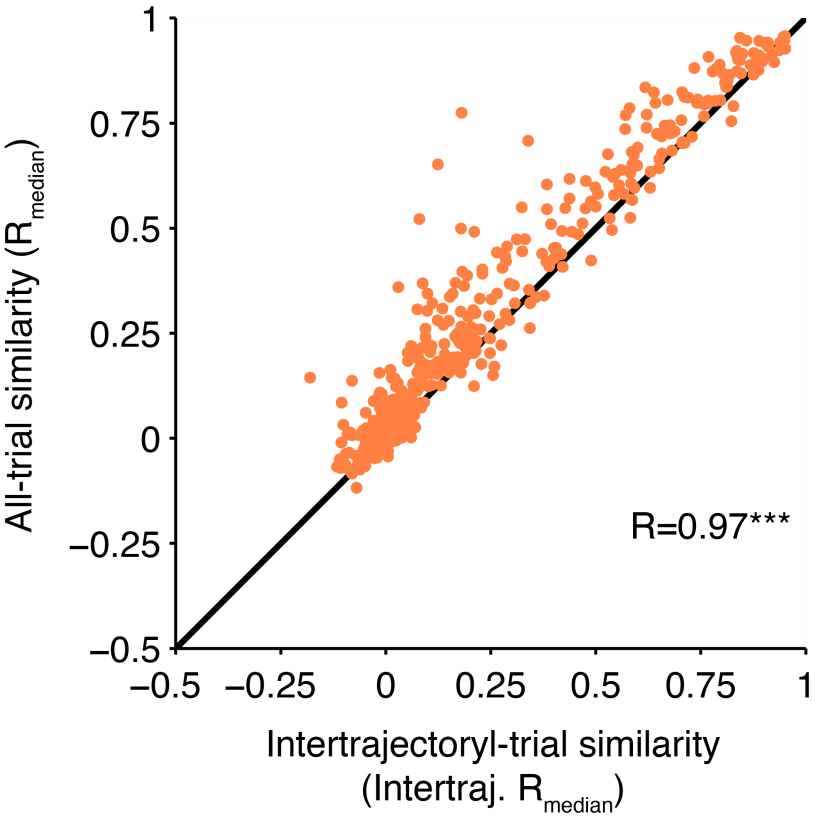
**

**Supplementary Figure 3. PFC All-trial similarity is correlated with Inter-trajectory-trial similarity.**

All-trial similarity (R_median_) is the median of Pearson’s correlation for all pair of trials. Intertrajectory-trial similarity (Intertraj. R_median_) is the median of Pearson’s correlation for all pairs of trials from different trajectories. PFC cells *N* = 556.

**
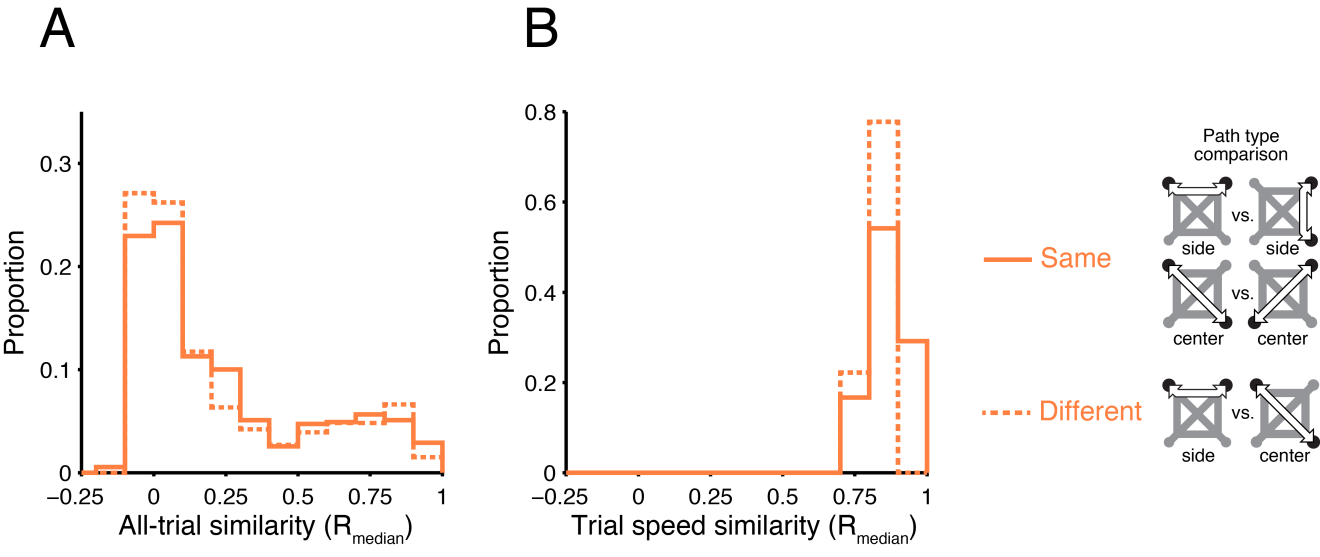
**

**Supplementary Figure 4. PFC firing is similar within and across trajectories with different lengths.**

**A.** R_median_ distribution for different path types for PFC units (same n=549 and different n=332). Kolmogorov-Smirnov and Wilcoxon rank-sum tests: not significant.

**B.** R_median_ distribution of trial speed similarity for days used to calculate firing similarity in A (same n=24 and different n=9). Kolmogorov-Smirnov and Wilcoxon rank-sum tests: not significant.

“Same” indicates comparisons between side with side or center with center trials. “Different” indicates comparisons between side and center trials.


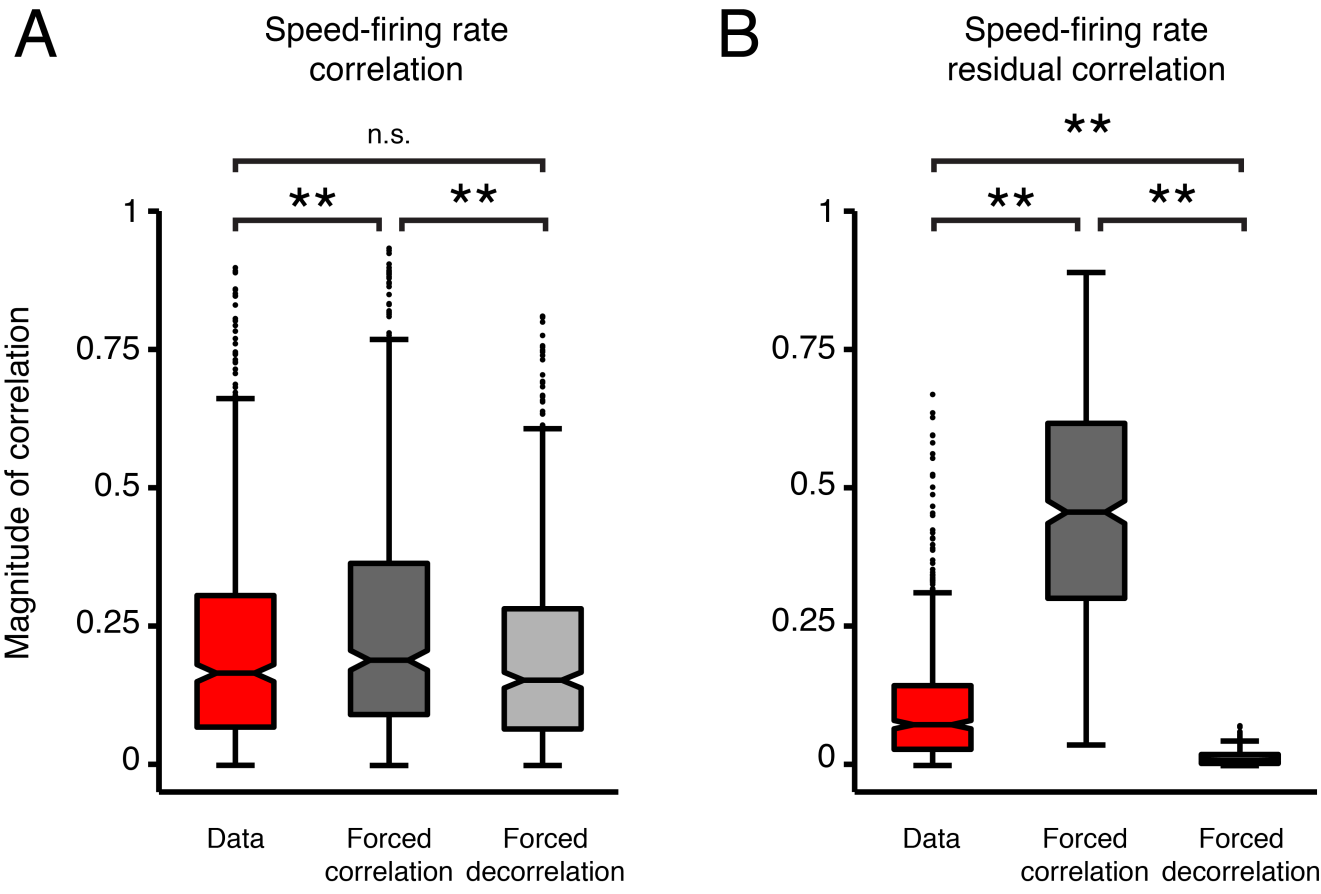


**Supplementary Figure 5. PFC firing cannot be explained by speed alone.**

**A.** Box plots of correlation magnitude between firing rate and animal speed of PFC cells (*N* = 556) for observed data (red), data where firing rate and speed were forced to be correlated for each trial bin (dark gray) and data where firing rate and speed were decorrelated for each trial bin (light gray). Observed firing rate-speed correlation magnitude was not significantly different from that of the decorrelated data set (red versus light gray). Forced correlation was achieved by sorting both firing rate and speed in descending order for data in each trial phase bin. This was done across all trials on the same trajectory. This served as a control to determine the expected maximum correlation magnitude when firing rate and speed are correlated while preserving the relationship between trajectory and trial phase related firing rate changes. Similarly, decorrelation was achieved by randomly permuting firing rate for data in each trial phase bin across trials on the same trajectory. This was a control to determine the expected correlation magnitude when firing rate and speed are decorrelated for each trial phase while preserving the relationship between trajectory and trial phase related firing rate changes. Wilcoxon rank-sum test: n.s. not significant and ** p<0.01.

**B.** Box plots of residual correlation magnitude between firing rate and animal speed for observed data (red), data in which firing rate and speed were forced to be correlated for each trial bin (dark gray) and data in which firing rate and speed were decorrelated for each trial bin (light gray). The distribution of firing rate-speed residual correlation magnitude is significantly lower than that of the correlated data set and higher than the decorrelated data set. Residuals were calculated by subtracting the mean values from data in each trial phase for all trials on the same trajectory. Wilcoxon rank-sum test: n.s. not significant and ** p<0.01.

**
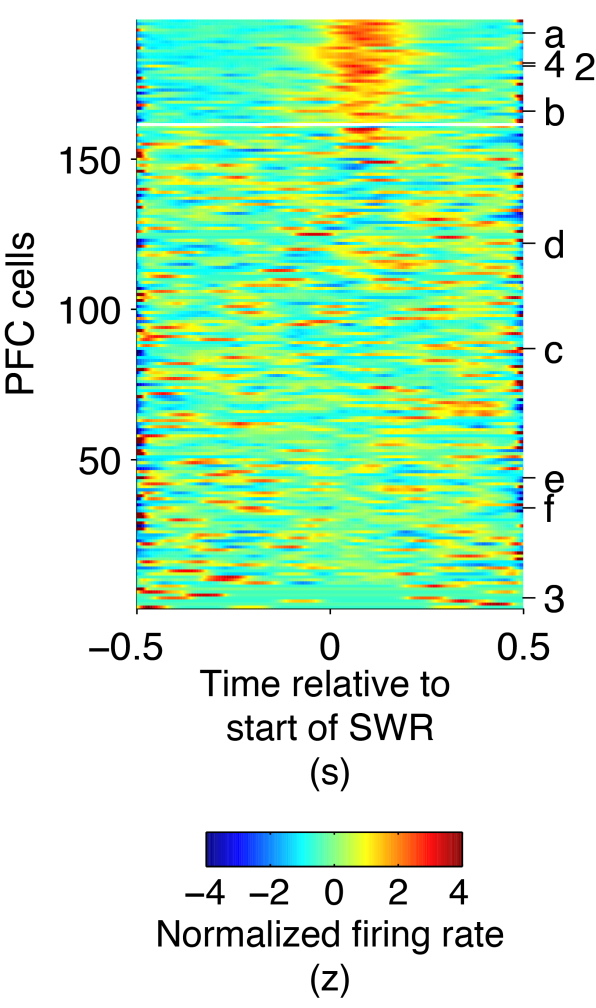
**

**Supplementary Figure 6. SWR aligned firing of PFC path-preferring cells.**

SWR aligned normalized firing rate for path-preferring PFC cells. Rows sorted by SWR modulation significance. Cells above the white line show significantly excitation (p<0.05) during path location SWRs. Example PFC cells from Fig. 1 (cells 2-4) and Fig. 4 (cells a-f) are indicated.


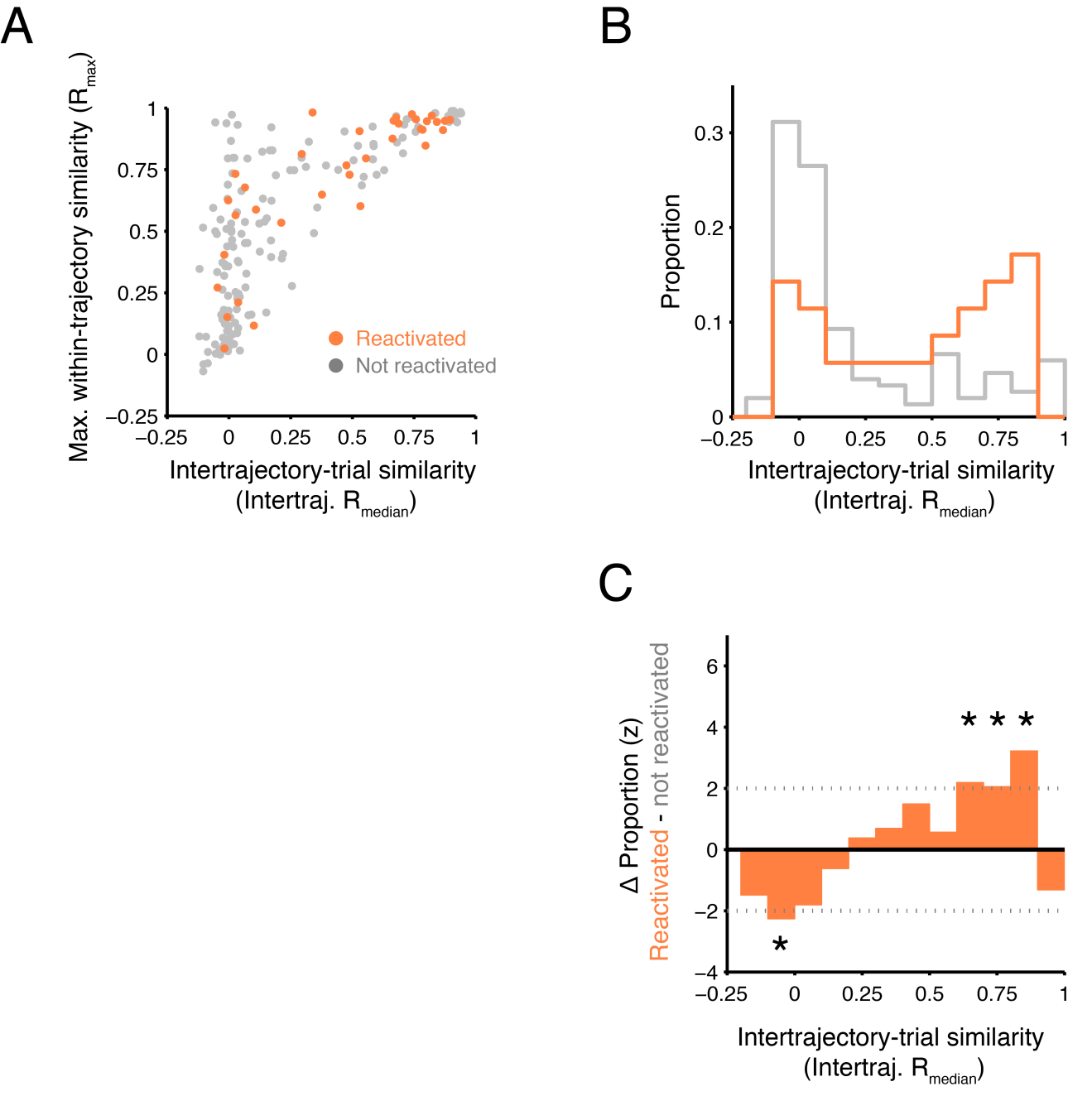


**Supplementary Figure 7. SWR reactivated path-preferring PFC cells show higher activity similarity across different trajectories.**

**A.** Replication of analyses in Fig. 5 with Intertraj. R_median_. Scatter of R_max_ and Intertraj. R_median_ for SWR reactivated and non-reactivated PFC cells.

**B.** Distribution of Intertraj. R_median_. Kolmogorov–Smirnov test: ***p<0.05.

**C.** Difference in the Intertraj. R_median_ distributions between path SWR reactivated and not reactivated PFC cells normalized using a permutation test (see Methods). Dotted lines indicate ±2 S.D..

**
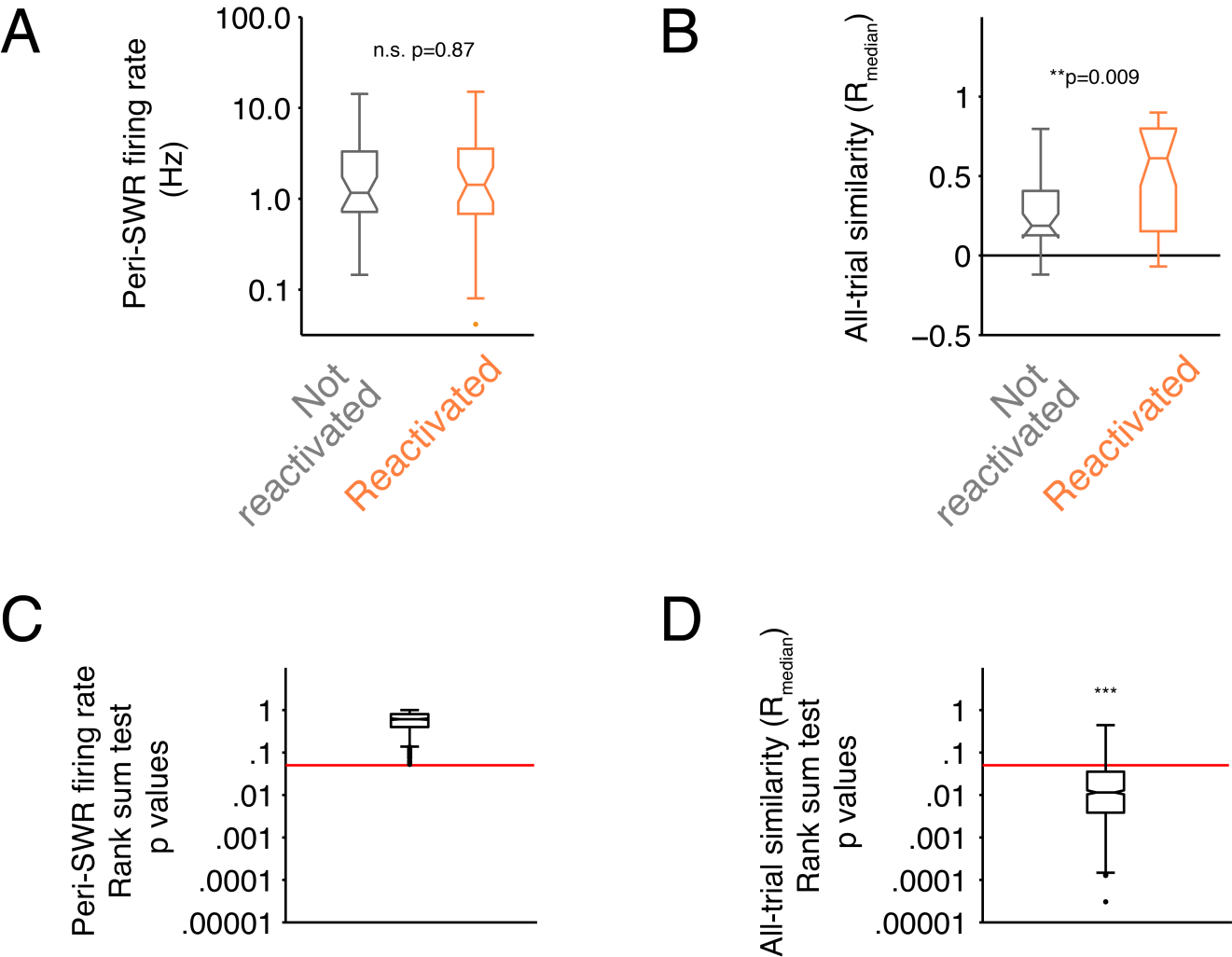
**

**Supplementary Figure 8. Higher all-trial similarity of SWR reactivated PFC cells compared with non-reactivated cells remains after matching peri-SWR firing rates.**

**A.** Boxplot of peri-SWR firing rates from an example resampled dataset where PFC cells from the non-reactivated group (gray) were selected to match the peri-SWR firing rates and the number of cells (n=35) from the reactivated group (orange).

**B.** Boxplot of corresponding all-trial firing similarity (R_median_) for resampled data in A. SWR reactivated cells (orange) show higher all-trial similarity compared with non-reactivated cells (gray) after matching peri-SWR firing rate.

**C.** Boxplot of Wilcoxon rank sum test p values for 1000 peri-SWR firing rate matched resamples. Red line indicates p=0.05.

**D.** Boxplot of Wilcoxon rank-sum test p values for differences in all-trial similarity for the corresponding 1000 resamples in B.

Binomial test for observed vs. expected proportion of resampled datasets with a significant difference (p<0.05, 5%) is ***p<10^-4^. Red line indicates p=0.05.

**
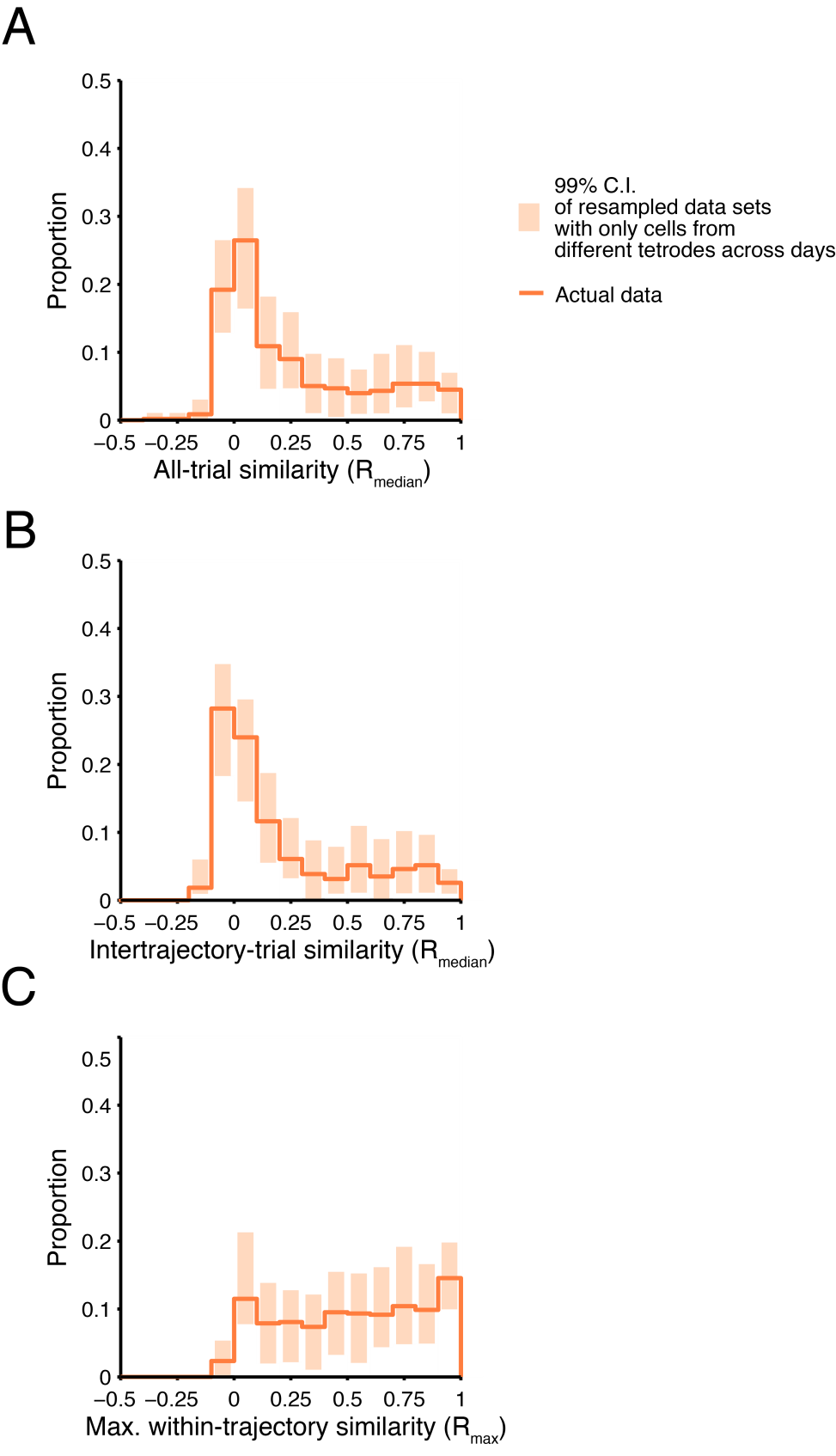
**

**Supplementary Figure 9. Distribution of trial similarity measures remains similar for data sets with unique cells.**

**A-C.** Trial firing similarity distributions for actual data (line) and 99% confidence interval (shading) of resampled data sets (*N* = 1000) with only unique cells. For each resample, each tetrode only contributed one day’s worth of cells for each animal. The actual data lies in the C.I of the unique cell data sets.

**
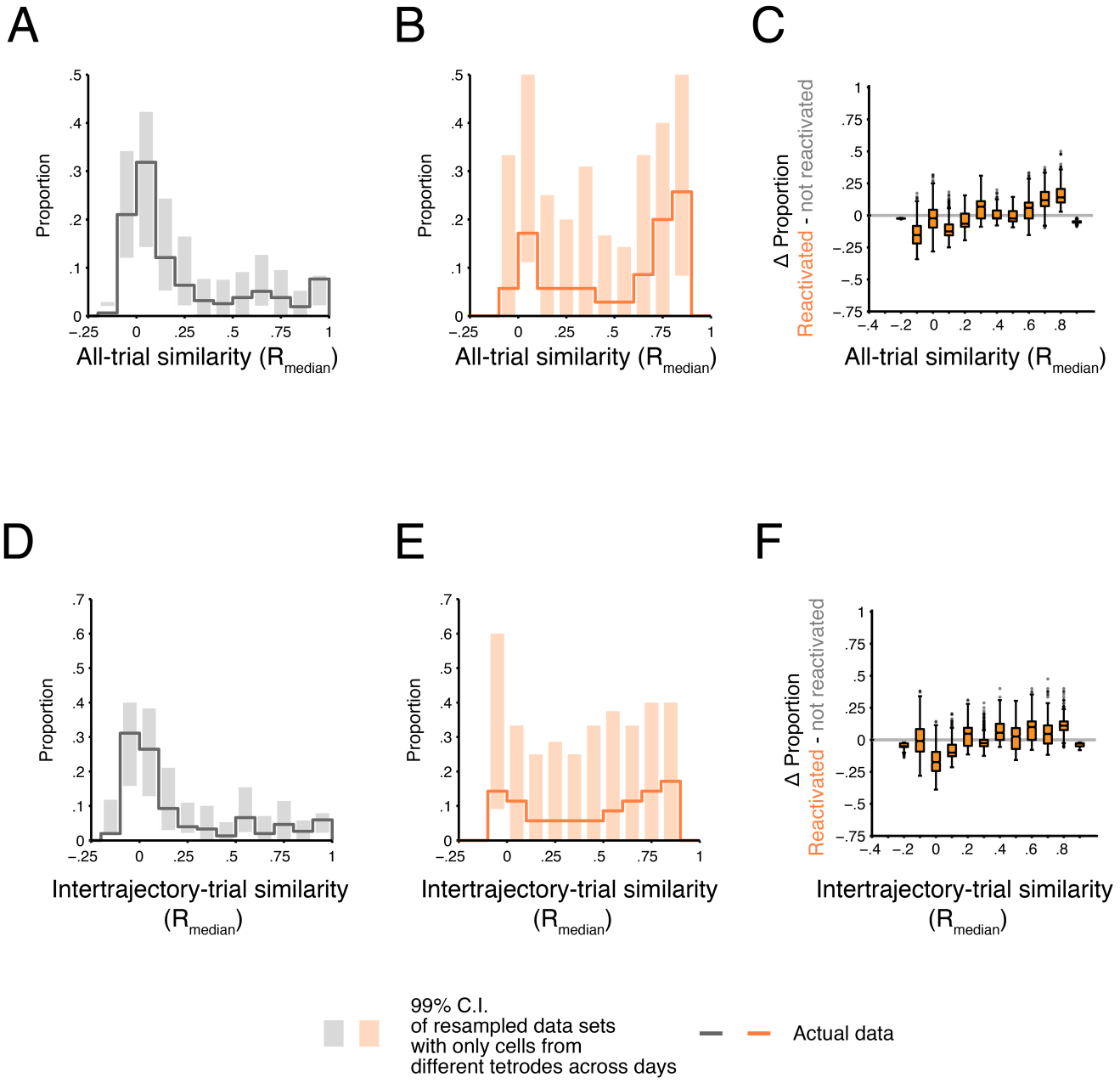
**

**Supplementary Figure 10. Higher trial similarity observed in the reactivated PFC population in unique-cell control data sets.**

**A-B and D-E.** Trial firing similarity distributions for actual data (line) and 99% confidence interval (shading) of resampled data sets (*N* = 1000) with only unique cells. For each resample, each tetrode only contributed one day’s worth of cells for each animal. The actual data lies in the C.I of the unique cell data sets.

**A and D.** Non SWR-reactivated PFC cells.

**B and E.** Reactivated cells.

**C and F.** Difference in the distribution between non-reactivated and reactivated cells for each of the 1000 resamples. Positive values indicate a greater proportion observed in the reactivated compared with the non-reactivated population.

**A-C.** All-trial similarity.

**D-F.** Intertrajectory-trial similarity.


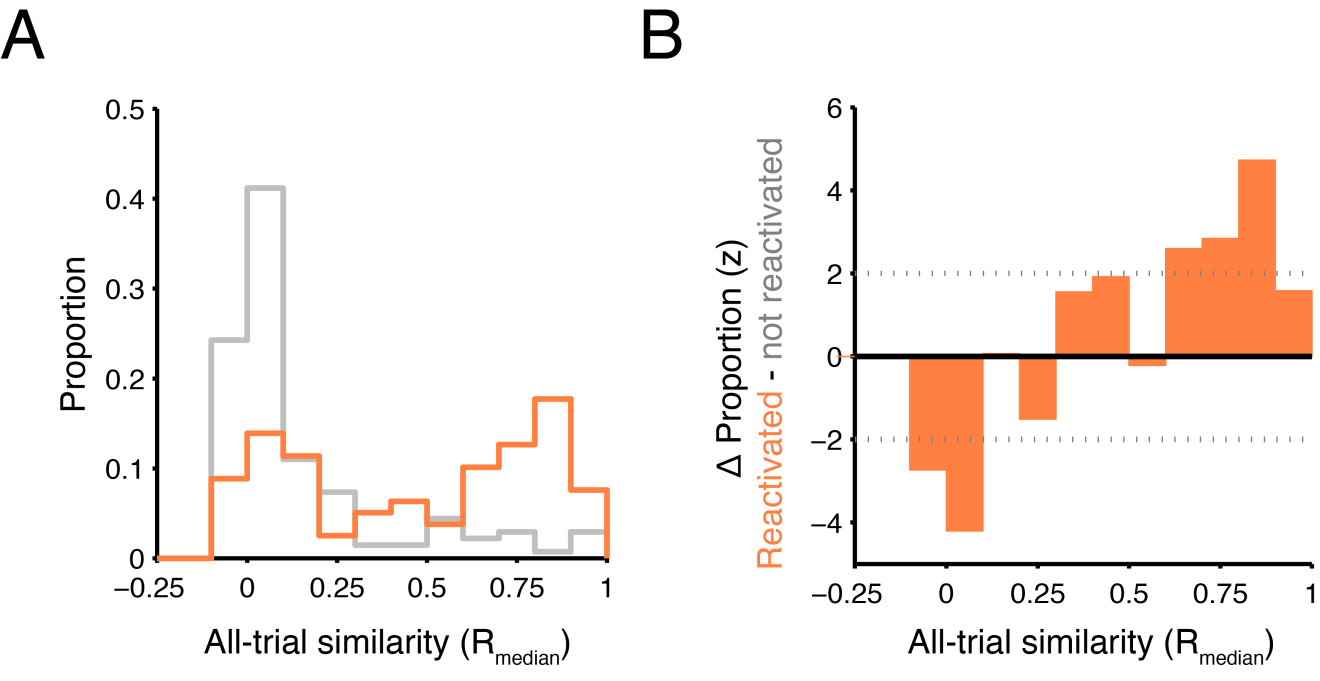


**Supplementary Figure 11. Enrichment of PFC cells with high R_median_ remains when all SWRs were included in calculating SWR modulation.**

A. Distribution of R_median_ distributions for SWR modulated (orange) and not modulated (gray) PFC cells. Kolmogorov–Smirnov test: ***p<10^-3^.

B. Difference in the R_median_ distributions between path SWR reactivated and not reactivated PFC cells normalized using a permutation test.


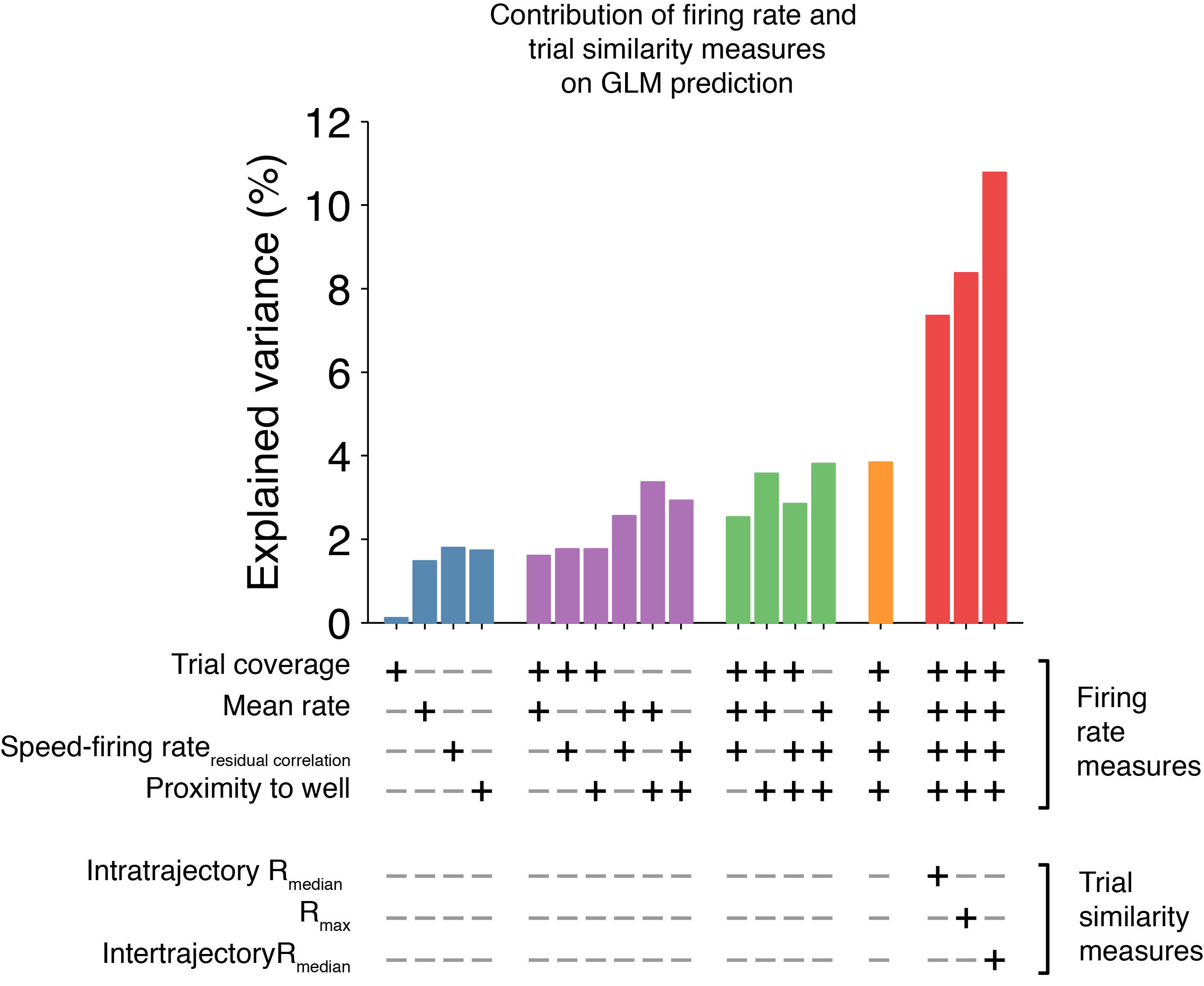


**Supplementary Figure 12. Trial similarity measures contribute more than trial firing measures for predicting SWR modulation.**

Explained variance was calculated for GLMs with a combination of variables to predict SWR modulation. The inclusion of trial similarity measures greatly improved the prediction.

**
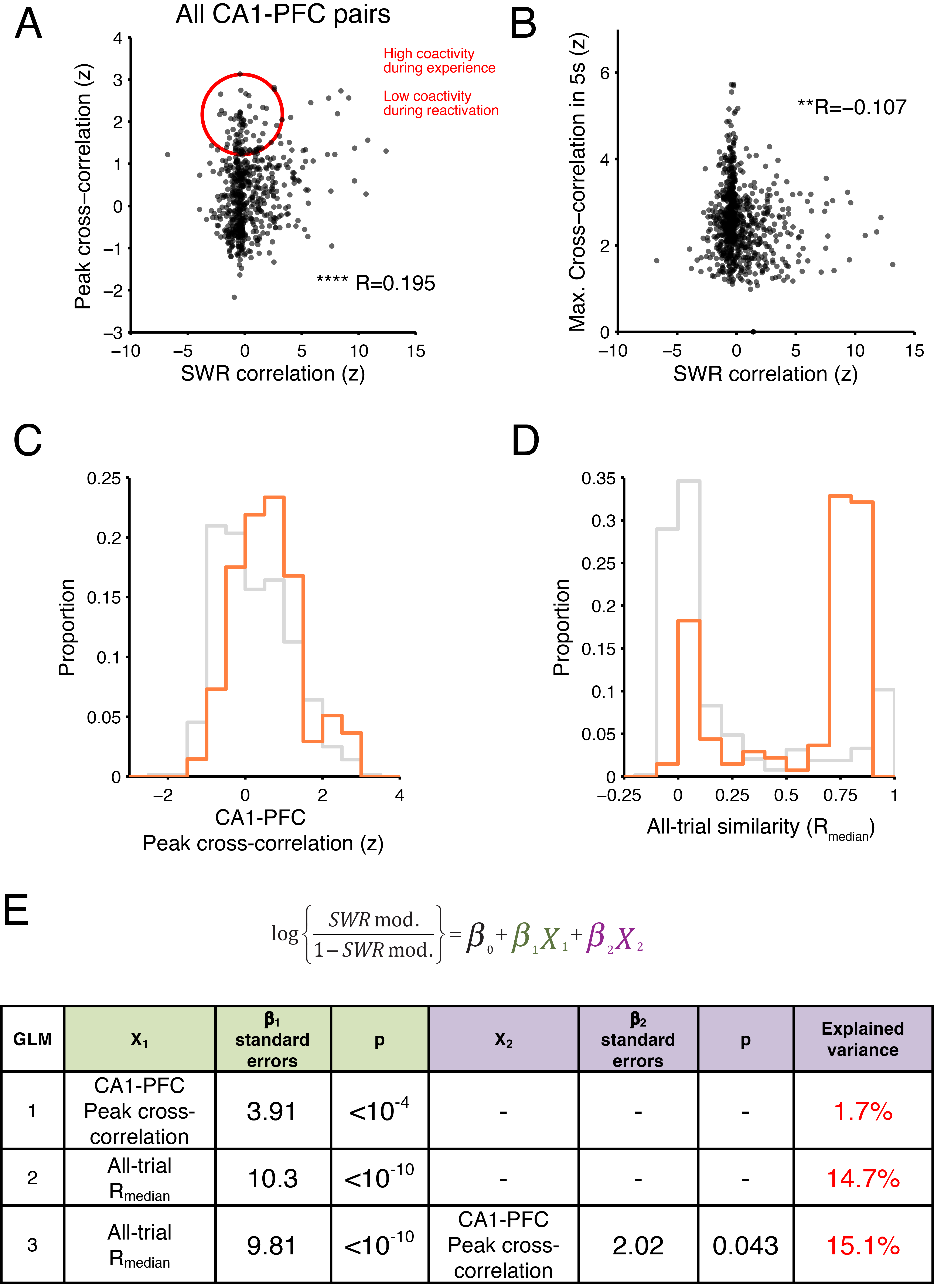
**

**Supplementary Figure 13. CA1-PFC coactivity during ongoing experience weakly explains coactivity during reactivation.**

**A.** Peak ongoing activity cross-correlation is weakly correlated with SWR activity correlation.

**B.** Maximum activity cross-correlation is weakly correlated with SWR activity correlation.

Hippocampal-PFC cell pairs what were coactivity during ongoing experience but were not reactivated together are circles in red. We computed coactivity between CA1-PFC cell pairs, during ongoing experience, we used the same subset of path-active PFC cells and calculated their spike cross-correlation over a 5 second window for times when the rat is on the path. For the peak cross-correlation, we took the z-scored values in a 100ms window centered on 0s lag as the measure of peak cross-correlation. For the maximum cross-correlation, we took the maximum value in the 5 second window. We calculated the maximum cross-correlation to account for the sequential nature of SWR reactivation, where CA1-PFC cells that were coactive on a time-scale longer than 100ms during behavior could be reactivated together. A high cross-correlation value indicates coactivity between CA1-PFC cell pairs during ongoing experience. To compute coactivity during SWRs, we then calculated the correlation the spike counts of the same pairs of CA1-PFC cells in a 200ms aligned to the start of SWRs. The correlation value is normalized by subtracting the mean and dividing by the standard deviation of the correlation values for 5000 shuffles where the identities of the reactivation events are permuted. A high correlation z indicates a CA1-PFC cell pair has coordinated firing during SWRs. ****p<0.001 and **p<0.005.

**C.** Distribution of peak cross-correlation for CA1-Not reactivated PFC pairs (gray) and CA1-Reactivated PFC pairs (orange).

**D.** Distribution of All-trial similarity (R_median_) for CA1-Not reactivated PFC pairs (gray) and CA1-Reactivated PFC pairs (orange). The R_median_ value refers to the R_median_ for the PFC cell in each cell pair.

**E.** All-trial similarity was a better predictor than peak cross-correlation for predicting whether the PFC cell in a CA1-PFC cell pair was reactivated during SWRs. A Generalized Linear Model (GLM) with one or two predictors and a logistic link function was used to predict PFC participation in reactivation, which was modeled as a binomial distribution (0 for non-reactivated and 1 for reactivated). The magnitude and significance of β for the predictor/s and the explained variance of the model is shown. βs are expressed as standard errors to allow comparison between predictors.


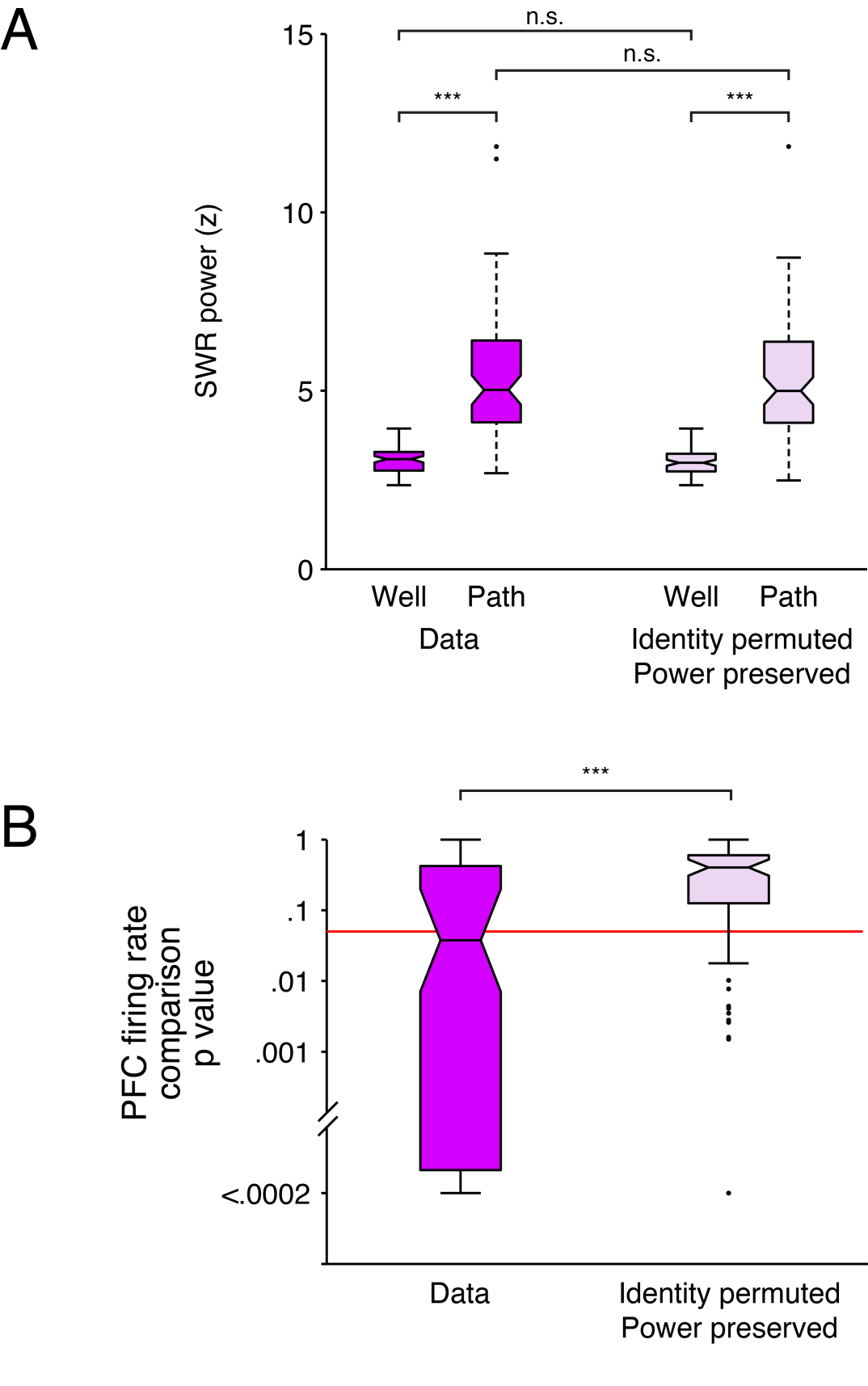


**Figure. S14. SWR power cannot explain PFC modulation.**

**A.** Boxplot of median SWR power for groups of reactivation events used to compare PFC modulation (purple, *N* = 87 pairwise comparisons). SWR reactivation events for Well location representations were lower in power compared with those for Path location representations. A permuted data set (light purple) was generated by sampling with replacement (*N* = 1000 for each pairwise comparison) where the power distribution and the number of events for each group was matched but the identities of the reactivation event (Well or Path) were permuted. Kruskall-Wallis test: n.s. not significantly and *** p<0.001.

**B.** Boxplot of p-values for firing rate comparisons between Well-Path reactivation events (purple) and the permuted data set from A (light purple). Firing rate comparisons for the power preserved but identity-permuted data set contained a lower proportion of significant differences (red line, p<0.05). Wilcoxon rank-sum test: *** p<0.001.
